# Comparison of broad-spectrum anti-Pseudomonal beta-lactam antibiotics versus targeted therapy for neutropenic patients with methicillin-susceptible *Staphylococcus aureus* bloodstream infections

**DOI:** 10.1017/ash.2025.179

**Published:** 2025-05-13

**Authors:** Lauren C. Russell, Jeff Klaus, Miguel A. Chavez, Erik R. Dubberke, Tamara Krekel

**Affiliations:** 1 Deparment of Pharmacy, Barnes-Jewish Hospital, Saint Louis, MO, USA; 2 Department of Medicine, Division of Infectious Diseases, Washington University School of Medicine in St. Louis, Saint Louis, MO, USA

## Abstract

The safety of the utilization of methicillin-susceptible *Staphylococcus aureus* (MSSA)-targeted therapy for the treatment of MSSA bloodstream infections in the setting of neutropenia is not well studied. This single-center, retrospective cohort study of 40 patients found no significant difference in clinical outcomes between broad-spectrum anti-Pseudomonal and MSSA-targeted beta-lactam therapy.

## Introduction

*Staphylococcus aureus* bloodstream infection (BSI) can result in serious morbidity and mortality, especially in neutropenic patients. Up to 8.4% of patients with febrile neutropenia have a *S. aureus* BSI, with mortality ranging from 15–45% at 3 months.^1–3^ Narrow-spectrum anti-staphylococcal beta-lactam therapy is considered standard of care for the treatment of methicillin-susceptible *S. aureus* (MSSA) BSI; however, standard of care for patients with febrile neutropenia is treatment with broad-spectrum anti-Pseudomonal beta-lactam therapy.^1,4–6^ A prior study found no significant difference in outcomes when comparing MSSA-targeted beta-lactam therapy to broad-spectrum anti-Pseudomonal beta-lactam therapy or combination therapy in neutropenic patients with MSSA BSI. Notably, the study did not describe therapy escalation or incidence of new gram-negative infections within the targeted therapy group, which is of interest with losing gram-negative coverage.^7^ The purpose of our study was to compare broad-spectrum, anti-Pseudomonal beta-lactam therapy (broad-spectrum therapy) to MSSA-targeted beta-lactam therapy (targeted therapy) during the period of neutropenia to determine if targeting therapy prior to neutrophil recovery is safe.

## Methods

### Study design and population

This retrospective, single-center, cohort study included adult patients admitted to an oncology service at Barnes-Jewish Hospital with an index MSSA-positive blood culture between 6/1/2018 and 1/31/2024. Patients were included if they were neutropenic within 24 h of the index MSSA-positive blood culture. Patients were excluded if they had positive identification of any organism on culture or polymerase chain reaction from any source within 2 weeks prior to the index MSSA-positive blood culture except for MSSA, gram-positive organisms predicted to be covered by an antibiotic in both treatment groups, viruses, or *Candida* species in the respiratory tract or urine. Patients were also excluded if they received ≥24 h of vancomycin, linezolid, or daptomycin monotherapy while neutropenic, if they had antibiotics modified to targeted therapy and were concomitantly initiated on anti-Pseudomonal fluoroquinolone prophylaxis, if they received a combination of both therapies for ≥48 h, or if they did not receive either therapy for ≥24 h. The study was approved by the Washington University in St. Louis Institutional Review Board.

### Study outcomes

The primary composite outcome was assessed from index MSSA blood culture positivity to neutrophil recovery, or hospital discharge if neutrophil recovery did not occur during hospitalization, and consisted of patients who met at least one of the following: all-cause mortality, new or recurrent fever, or identification of a gram-negative organism from any site. Secondary outcomes also assessed during this time frame included the components of the primary outcome, time to new or recurrent fever, new intensive care unit (ICU) admission, reason for new ICU admission, ICU length of stay (LOS), broadening of therapy, reason for broadening of therapy, and duration of broad-spectrum anti-Pseudomonal beta-lactam therapy. Additional secondary outcomes included 30-day all cause-mortality, 90-day MSSA BSI recurrence, length of antibiotic therapy for MSSA BSI, overall hospital LOS, and *Clostridioides difficile* infection.

### Study definitions

Neutropenia was defined as an absolute neutrophil count (ANC) <500 cells/µL. The broad-spectrum therapy group received cefepime or meropenem for treatment of their MSSA BSI the entire time while neutropenic. The targeted therapy group included patients whose antibiotics were modified to oxacillin, cefazolin, or ceftriaxone for treatment of their MSSA BSI at any point while neutropenic. Broadening of therapy was defined as the use of a beta-lactam with a broader spectrum of activity as determined by a modified gram-negative spectrum score^8^ (Supplemental Table 1) or the addition of a systemic anti-Pseudomonal fluroquinolone or aminoglycoside. Modification to another MSSA-targeted beta-lactam did not count as broadening of therapy. A complete list of study definitions is available in the Supplement.

### Statistical analysis

A description of statistical analyses is available in the Supplement.

## Results

A total of 263 patients were screened for eligibility (Supplemental Figure 1). Of the 40 patients included, 27 (67.5%) had antibiotics modified to targeted therapy and 13 (32.5%) remained on broad-spectrum therapy while neutropenic.

No statistically significant differences existed between groups with regards to baseline characteristics except significantly more patients received meropenem initially in the broad-spectrum therapy group (38.5% vs. 7.4%, *P* = 0.027). Patients in the broad-spectrum therapy group had a numerically longer median duration of neutropenia after index MSSA blood culture positivity (7.6 days vs. 4.8 days, *P* = 0.130). The median time to targeting therapy was 1.9 days, 81.5% of patients had achieved BSI clearance at the time of targeting, and patients received a median of 2.3 days of targeted therapy while neutropenic (Table 1).

**Table 1.** Baseline patient/treatment characteristics

Characteristic	Broad-spectrum therapy group (N = 13)	Targeted therapy group (N = 27)	P value
Age, years, mean (± SD)	58.7 (± 12.6)	57.4 (± 13.8)	0.779
Weight, kg, median (IQR)	97.1 (77.5–105.7)	87.0 (72.6–102.5)	0.386
BMI, kg/m^2^, median (IQR)	32.2 (25.5–35.2)	28.8 (25.1–35.7)	0.942
Male sex, n (%)	9 (69.2)	11 (40.7)	0.091
CCI, median (IQR)	3 (2–6)	3 (2–6)	0.940
ICU at time of index MSSA-positive blood culture, n (%)	1 (7.7)	5 (18.5)	0.643
Fever within 24 h of index MSSA-positive blood culture, n (%)	10 (76.9)	25 (92.6)	0.307
Gram-positive organism identified within 2 weeks of index MSSA-positive blood culture, n (%)^1^	4 (30.8)	3 (11.1)	0.187
Initial broad-spectrum therapy, n (%)			0.027
Cefepime	8 (61.5)	25 (92.6)	
Meropenem	5 (38.5)	2 (7.4)	
Initial targeted therapy, n (%)			
Cefazolin	–	16 (59.3)	–
Oxacillin	–	5 (18.5)	–
Ceftriaxone	–	6 (22.2)	–
ID consult, n (%)	12 (92.3)	27 (100.0)	0.325
Source of infection, n (%)			
Catheter-related	6 (46.2)	16 (59.3)	0.435
Skin and skin structure	0	3 (11.1)	0.538
Respiratory	0	1 (3.7)	> 0.999
Enterocolitis	1 (7.7)	0	0.325
Unknown	6 (46.2)	7 (25.9)	0.284
Endocarditis after index MSSA-positive blood culture, n (%)	0	5 (18.5)	0.154
Bone/joint infection after index MSSA-positive blood culture, n (%)	1 (7.7)	1 (3.7)	> 0.999
Malignancy type, n (%)			
AML/MDS	5 (38.5)	5 (18.5)	0.246
ALL	1 (7.7)	2 (7.4)	> 0.999
Lymphoma	2 (15.4)	4 (14.8)	> 0.999
Chronic lymphoid malignancies	0	1 (3.7)	> 0.999
MM	2 (15.4)	9 (33.3)	0.286
Solid tumor	0	4 (14.8)	0.284
Other^2^	3 (23.1)	2 (7.4)	0.307
Causes of neutropenia, n (%)			
Chemotherapy	7 (53.8)	13 (48.1)	0.736
Allogeneic-HCT	3 (23.1)	3 (11.1)	0.370
Autologous-HCT	1 (7.7)	10 (37.0)	0.068
Underlying malignancy	2 (15.4)	1 (3.7)	0.242
Retainment of central line, n (%)	3 (23.1)	2 (7.4)	0.307
Time to removal of central line after index MSSA blood culture positivity, days, median (IQR)	2.1 (1.5-5.3)	2.3 (1.3-3.3)	0.812
Discharged prior to neutrophil recovery, n (%)	2 (15.4)	6 (22.2)	> 0.999
Duration of neutropenia after index MSSA blood culture positivity, days, median (IQR)	7.6 (4.0–19.3)	4.8 (4.0–8.2)	0.130
Duration of neutropenia after broad-spectrum therapy initiation, days, median (IQR)	7.0 (4.8–20.3)	5.6 (4.6–8.8)	0.214
Duration of neutropenia after broad-spectrum therapy initiation and before targeting, days, median (IQR)	–	2.5 (2.0–3.5)	–
Duration of neutropenia after targeting, days, median (IQR)	–	2.6 (1.4–6.2)	–
Time to targeting from index MSSA-positive blood culture, days, median (IQR)	–	1.9 (1.3-2.5)	–
Duration of targeted therapy while neutropenic, days, median (IQR)^3^	–	2.3 (1.3–4.0)	–
BSI clearance at time of targeting, n (%)	–	22 (81.5)	–
Fever within 24 h of targeting, n (%)	–	6 (22.2)	–

Abbreviations: ALL, acute lymphoblastic leukemia; AML, acute myeloid leukemia; BMI, body mass index; BSI, bloodstream infection; CCI, Charlson Comorbidity Index; HCT, hematopoietic cell transplantation; ICU, intensive care unit; IQR, interquartile range; MDS, myelodysplastic syndromes; MM, multiple myeloma; MSSA, methicillin-susceptible *Staphylococcus aureus.*

1 Gram-positive organisms identified: *Streptococcus* species (n = 9), *Clostridium perfringens* (n = 1).

2 Other malignancy types: chronic myelogenous leukemia (n = 1) and myeloproliferative neoplasms/disorders (n = 4).

3 Duration of targeted therapy while neutropenic includes duration of targeted therapy after re-targeting if patient was broadened and then received targeted therapy again prior to neutrophil recovery.

No statistical difference existed in the primary composite outcome between the broad-spectrum therapy and targeted therapy groups (38.5% vs. 22.2%, *P* = 0.451), which was met by experiencing new or recurrent fever in all patients. One patient in the broad-spectrum therapy group had a gram-negative organism identified on culture, compared to no patients in the targeted therapy group. This patient was receiving cefepime and had two gram-negative organisms, susceptible to cefepime, identified on blood culture the day after the index MSSA-positive blood culture. One patient in the targeted therapy group experienced a fatal intraparenchymal hemorrhage, deemed to be unrelated to infection. A shorter duration of broad-spectrum anti-Pseudomonal beta-lactam therapy was noted in the targeted therapy group (median 7.2 days vs. 2.3 days, *P* < 0.001). All other secondary endpoints were not statistically significant (Table 2).

**Table 2.** Primary and secondary outcomes

Outcome	Broad-spectrum therapy group (N = 13)	Targeted therapy group (N = 27)	P value
Primary composite outcome, n (%)^1^	5 (38.5)	6 (22.2)	0.451
All-cause mortality, n (%)	0	1 (3.7)	> 0.999
New or recurrent fever, n (%)	5 (38.5)	6 (22.2)^2^	0.451
Time to new or recurrent fever, days, mean (±SD)	3.5 (± 2.8)	4.1 (± 2.6)	0.713
Identification of gram-negative from any site, n (%)	1 (7.7)	0	0.325
New ICU admission, n (%)	2 (15.4)	2 (7.4)	0.584
Reason for ICU admission, n (%)			
Worsening infectious symptoms	1/2 (50.0)	0	> 0.999
Non-infection related	1/2 (50.0)	2/2 (100.0)	> 0.999
Broadening of therapy, n (%)	4 (30.8)	8 (29.6)	> 0.999
Broader-spectrum beta-lactam	2/4 (50.0)	7/8 (87.5)	0.236
Anti-Pseudomonal fluoroquinolone addition	0	1/8 (12.5)	> 0.999
Aminoglycoside addition	2/4 (50.0)	0	0.091
Reason for broadening of therapy, n (%)			
New infection	1/4 (25.0)	2/8 (25.0)	> 0.999
Worsening/persistent infectious symptoms^3^	2/4 (50.0)	4/8 (50.0)	> 0.999
Unknown	1/4 (25.0)	1/8 (12.5)	> 0.999
Other^4^	0	1/8 (12.5)	> 0.999
30-day all-cause mortality, n (%)	4 (30.8)	2 (7.4)	0.075
90-day MSSA recurrence, n (%)	0/9	0/24	–
Duration of blood culture positivity, days, median (IQR)	1.1 (1.0–1.8)	1.1 (1.0–2.1)	0.806
LOS, days, median (IQR)	24.7 (13.9-33.6)	19.0 (13.7-26.3)	0.199
ICU LOS from index MSSA blood culture positivity to ANC recovery, mean (± SD)	7.6 (± 6.1)	2.4 (± 1.9)	0.062
Length of antibiotic therapy for BSI, days, mean (± SD)	29.2 (± 14.6)	31.9 (± 12.3)	0.530
30-day readmission, n (%)	5/9 (55.6)	4/24 (16.7)	0.073
*C. difficile* infection after index MSSA-positive blood culture during admission, n (%)	0	1 (3.7)	> 0.999
Duration of broad-spectrum anti-Pseudomonal beta-lactam therapy after index MSSA-positive blood culture while neutropenic, days, median (IQR)^5^	7.2 (3.8–19.1)	2.3 (1.1–3.6)	< 0.001

Abbreviations: BSI, bloodstream infection; ICU, intensive care unit; IQR, interquartile range; SD, standard deviation; LOS, length of stay; MSSA, methicillin-susceptible *Staphylococcus aureus.*

1 The primary composite outcome was assessed from index MSSA blood culture positivity to neutrophil recovery, or hospital discharge if neutrophil recovery did not occur during the hospital stay, and consisted of patients who met at least one of the following: all-cause mortality, new or recurrent fever, or identification of a gram-negative organism from any site. Patients meeting more than one component could only meet the primary composite outcome once.

2 One patient met the primary outcome (new or recurrent fever) while on both broad-spectrum therapy and targeted therapy (overlap of therapies <48 h).

3 All patients broadened for worsening/persistent infectious symptoms had a fever at the time of broadening.

4 Other reason for broadening of therapy: ciprofloxacin added to oxacillin due to intention to switch to targeted therapy with anti-Pseudomonal fluoroquinolone concomitantly that was delayed to the next day; patient received 3 doses of ciprofloxacin prior to switch to ceftriaxone monotherapy.

5 Duration of broad-spectrum therapy after index MSSA-positive blood culture while neutropenic includes duration of broadened therapy if patient broadened to a broad-spectrum anti-Pseudomonal beta-lactam.

## Discussion

We found similar outcomes when broad-spectrum anti-Pseudomonal beta-lactam therapy was modified to MSSA-targeted beta-lactam therapy prior to neutrophil recovery in an oncology patient population. Importantly, no patients in the targeted therapy group had a new gram-negative identified on culture during their duration of neutropenia as loss of gram-negative, especially Pseudomonal, coverage is the primary concern with modification to targeted therapy in patients with neutropenia. The time to targeted therapy after identification of MSSA on blood culture was a median (IQR) of 1.9 (1.3–2.5) days in our study, demonstrating modification of antibiotics to targeted therapy can be done early in the treatment course.

Patients who were continued on broad-spectrum therapy had a numerically higher rate of 30-day all-cause mortality and readmission and an increased ICU LOS from index MSSA blood culture positivity to ANC recovery, though none of these differences were statistically significant. Differences in baseline characteristics, including 20% more patients with acute myeloid leukemia/myelodysplastic syndromes and significantly increased empiric meropenem use in the broad-spectrum therapy group, suggest this group was potentially a more unstable, complicated population compared to the targeted therapy group.

This study has several limitations, the main one being the small sample size, which may have reduced the ability to detect a statistical difference between groups. Data was collected via manual retrospective chart review and limited to the accuracy of documentation. Patients included in this study had a short duration of neutropenia after index MSSA blood culture positivity overall, and the median duration of neutropenia while on targeted therapy was only 2.3 days, which limits the generalizability to patients who have longer expected durations of neutropenia. Strengths of this study include the strict eligibility criteria that limited confounding variables and selected a truly high-risk patient population and assessment of safety outcomes, including broadening of therapy and identification of new gram-negative organisms, which were absent from prior literature.^7^

In our study sample, worse outcomes were not found after modification of empiric broad-spectrum anti-Pseudomonal beta-lactam therapy to MSSA-targeted beta-lactam therapy in neutropenic patients with MSSA BSI. Modification of broad-spectrum therapy to target MSSA BSI in neutropenic patients prior to neutrophil recovery should be considered.

## Supporting information

Russell et al. supplementary material Russell et al. supplementary material

## Data Availability

Research data are not shared.

## Supplementary material

For supplementary material accompanying this paper visit http://doi.org/10.1017/ash.2025.179. click here to view supplementary material
