# Supplementary material for: Comparison of broad-spectrum anti-Pseudomonal beta-lactam antibiotics versus targeted therapy for neutropenic patients with methicillin-susceptible Staphylococcus aureus bloodstream infections
Source: Antimicrob Steward Healthc Epidemiol. 2025 May 13;5(1):e108. doi: 10.1017/ash.2025.179 (PMC12086594; doi:10.1017/ash.2025.179)
Supplement: Russell et al. supplementary material [file S2732494X25001792sup001.pdf]

Comparison of Broad-spectrum Anti-Pseudomonal Beta-lactam Antibiotics versus Targeted Therapy for Neutropenic Patients with Methicillin-susceptible *Staphylococcus aureus* Bloodstream Infections

Supplementary Material

Study definitions

- Neutropenia: absolute neutrophil count (ANC) < 500 cells/µL.
- Neutrophil recovery: ANC ≥ 500 cells/µL for ≥ 48 hours.
- Index MSSA-positive blood culture: the first blood culture that was positive for MSSA and occurred within 24 hours of a time point in which the patient met criteria for neutropenia.
- Index MSSA blood culture positivity: time zero for the primary outcome; defined as the date and time of identification of MSSA, which was done via Verigene® in the majority of patients (all but two patients; one in each group), and via penicillin-binding protein 2a (PBP2a) testing in patients without Verigene® identification. Identification of MSSA was confirmed with susceptibility testing via Kirby-Bauer disk diffusion for all patients.
- Clearance of BSI: two negative blood cultures after the index MSSA-positive blood culture, if available; if only one repeat negative blood culture was drawn, this counted as clearance.
- Recurrence of MSSA BSI: clearance of MSSA on blood culture followed by isolation of MSSA on repeat blood culture.

- Broad-spectrum anti-Pseudomonal beta-lactam therapy group (broad-spectrum therapy group): received cefepime or meropenem for treatment of their MSSA BSI the entire time while neutropenic.
- MSSA-targeted beta-lactam therapy group (targeted therapy group): included patients whose antibiotics were modified to oxacillin, cefazolin, or ceftriaxone for treatment of their MSSA BSI at any point while neutropenic.
- Broadening of therapy: the use of a beta-lactam with a broader spectrum of activity as determined by modified gram-negative spectrum score (Supplemental Table 1) or the addition of a systemic anti-Pseudomonal fluroquinolone or aminoglycoside. Modification to another MSSA-targeted beta-lactam did not count as broadening of therapy.
- Time to targeting: the time from the index MSSA blood culture positivity to the time of administration of targeted therapy.
- Fever: a one-time temperature of ≥ 38°C.
- Defervescence: a temperature of ≤ 37.6°C for ≥ 24 hours after meeting the criteria for fever.
- Recurrence of fever: fever after defervescence.

Patients were not required to be febrile for inclusion; therefore, patients could have an outcome of new fever if they were afebrile within 24 hours before and after of the index MSSA-positive blood culture or recurrent fever if they were febrile within 24 hours of the index MSSA-positive blood culture, met criteria for defervescence, and then met criteria for recurrent fever.

Statistical analysis

The primary outcome was compared between groups with the Fisher’s Exact test. Baseline characteristics and secondary outcomes were assessed with either Fisher’s Exact or Chi square tests for categorical variables, as appropriate, or Student’s t-test or Mann Whitney-U test for continuous variables, as appropriate. Statistical analyses were performed using IBM SPSS software, version 22, and a two-sided p-value of < 0.05 was considered statistically significant for all tests.

Supplemental Figure 1: Flow Diagram

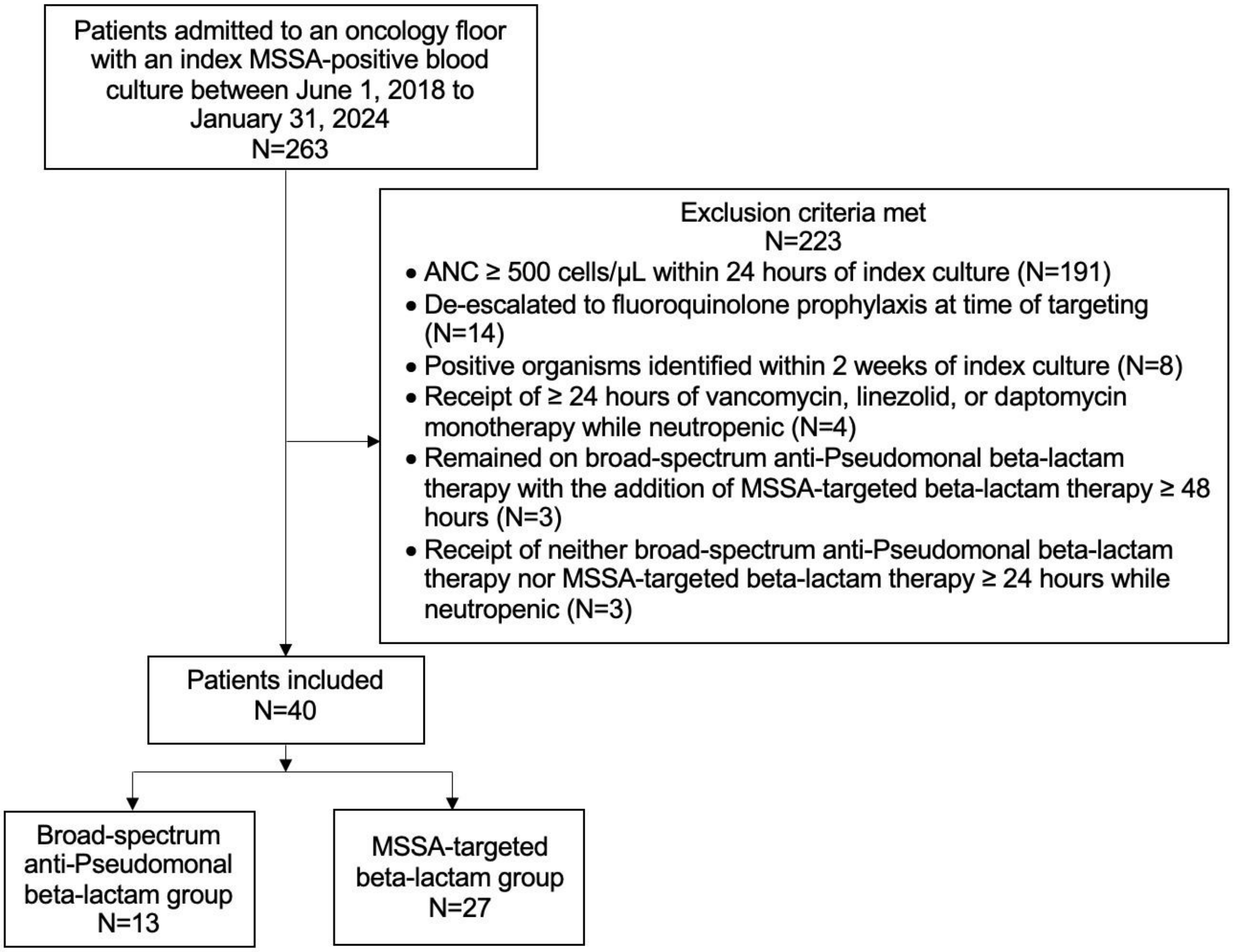

Supplemental Table 1: Modified Gram-negative Spectrum Score

		Wild Type					Acquired Resistance	
**Antibiotic**	**Spectrum Score**	*Moraxella catarrhalis, Haemophilus influenzae*	*Escherichia coli, Klebsiella pneumoniae*	*Enterobacter cloacae, Klebsiella aerogenes, Citrobacter freundii*	*Pseudomonas aeruginosa*	*Acinetobacter calcoaceticus-baumannii* complex	ESBL	CRE
Cefadroxil	0.75	0	0.75	0	0	0	0	0
Cefazolin	0.75	0	0.75	0	0	0	0	0
Cephalexin	0.75	0	0.75	0	0	0	0	0
Amoxicillin	1.5	1	0.5	0	0	0	0	0
Ampicillin	1.5	1	0.5	0	0	0	0	0
Amoxicillin/clavulanate	1.75	1	0.75	0	0	0	0	0
Cefuroxime	1.75	1	0.75	0	0	0	0	0
Cefoxitin	1.75	1	0.75	0	0	0	0	0
Cefixime	2	1	1	0	0	0	0	0
Cefdinir	2	1	1	0	0	0	0	0
Ceftriaxone	2	1	1	0	0	0	0	0
Ceftaroline	2	1	1	0	0	0	0	0
Ampicillin/sulbactam	2.5	1	0.75	0	0	0.75	0	0
Aztreonam	2.75	1	1	0	0.75	0	0	0
Ceftazidime	3.5	1	1	0	1	0.5	0	0
Ertapenem	4	1	1	0	0	0	1	0
Piperacillin/tazobactam	4.5	1	1	1	1	0.5	0	0
Cefepime	4.5	1	1	1	1	0.5	0	0
Ceftolozane/tazobactam	5	1	1	1	1	0	1	0
Imipenem/cilastatin	5.5	1	1	1	1	0.5	1	0
Meropenem	5.5	1	1	1	1	0.5	1	0

Ceftazidime/avibactam	6.5	1	1	1	1	0.5	1	1
Imipenem/cilastatin/relebactam	6.5	1	1	1	1	0.5	1	1
Meropenem/vaborbactam	6.5	1	1	1	1	0.5	1	1
Cefiderocol	7	1	1	1	1	1	1	1

Abbreviations: CRE, carbapenem-resistant Enterobacterales; ESBL, extended-spectrum beta-lactamase
